# Patient-Reported Outcomes of Antidepressant Drug Therapy: A Comparative Analysis

**DOI:** 10.1192/j.eurpsy.2025.1343

**Published:** 2025-08-26

**Authors:** P. Marinovic, J.-C. Radermacher, J. Stingl, N. Bozina, A. Mihaljevic-Peles, M. Zivkovic

**Affiliations:** 1 Clinic of psychiatry and psychological medicine, University Hospital Center Zagreb, Zagreb, Croatia; 2 University Hospital RWTH Aachen, Aachen, Germany; 3 University of Zagreb, School of medicine, Zagreb, Croatia

## Abstract

**Introduction:**

Patient-reported outcomes are becoming increasingly important in the evaluation of antidepressant drug therapy. They capture the patient’s perspective on their symptoms, treatment experiences, and overall well-being, unlike traditional clinical measures that focus only on objective assessments (clinical scales measuring symptom reduction and psychological changes). These outcomes provide a more comprehensive understanding of the impact of antidepressants on daily living.

**Objectives:**

The main objective of the study was to determine patient-reported outcomes of antidepressant drug therapy.

**Methods:**

300 patients from three study centers were included - a geriatric hospital (Germany), a Specialized psychiatric clinic (Germany), Outpatient psychiatric clinic (Croatia). Patients were assessed with the same questionnaire that consisted of two parts - the first one included questions about the patient’s current state (possible symptoms) and the second one included questions about possible antidepressant side effects. The main inclusion criterium was continuous antidepressant therapy, and the main exclusion criterium was the inability to communicate via a questionnaire.

**Results:**

Comparative analysis of patient perceptions in an acute psychiatric, outpatient psychiatric and geriatric patient cohort showed all patient cohorts were adherent to taking their medication but differed significantly in the level of awareness of any information regarding their medication taking (why are they taking it, when, what is the prescribed dose, etc.) with geriatric patients being the least aware. The results also show other differences between the three cohorts, especially between the Croatian and German cohorts. An interesting difference is shown in the open question about medication side effects - 99 Croatian patients didn’t report having side effects, while only 31 patients from one German center and 71 from another German center answered that they had no side effects when asked openly. When asked specifically, patients from all three cohorts gave similar answers.

**Conclusions:**

Understanding patient-reported outcomes can be extremely beneficial, as antidepressant efficacy as well as side effect severity varies widely among individuals and affects adherence to treatment. Patient-reported outcomes are a great starting point for moving in the direction of patient-centered medicine and can help healthcare providers make more informed decisions regarding antidepressant drug therapy.

This study is a part of ArtiPro ERA PerMed (Multidisciplinary research projects on personalized medicine- development of clinical support tools for personalized medicine implementation) project. The main goal is to establish an artificial intelligence-based platform that integrates data from clinical research involving biomarkers as predictors of illness and treatment outcomes with a goal to identify robust multimodal biomarkers and outcomes for depression.

**Disclosure of Interest:**

None Declared

